# Corticosteroid Treatment Prevents Lipopolysaccharide-Induced Increase of ACE2 and Reduces Fibrin Degradation Products in Bronchoalveolar Lavage Fluid

**DOI:** 10.3389/fmed.2022.856891

**Published:** 2022-03-02

**Authors:** Roman Reindl-Schwaighofer, Farsad Eskandary, Johann Bartko, Andreas Heinzel, Bernd Jilma, Manfred Hecking, Christian Schoergenhofer

**Affiliations:** ^1^Department of Nephrology and Dialysis, Division of Medicine III, Medical University of Vienna, Vienna, Austria; ^2^Department of Clinical Pharmacology, Medical University of Vienna, Vienna, Austria

**Keywords:** randomized controlled trial, acute lung injury, Renin-Angiotensin-Aldosterone System, Angiotensin-Converting Enzyme 2, fibrin degradation products

## Abstract

The assessment of systemic corticosteroid effects on intrapulmonary disease biomarkers is challenging. This retrospective evaluation of a human endotoxemia model quantified ACE2 and fibrin degradation product (FDP) concentrations in bronchoalveolar lavage fluid (BALF) samples from a randomized, double-blind, placebo-controlled study (NCT01714427). Twenty-four healthy volunteers received either 2 × 40 mg intravenous dexamethasone or placebo. These doses were administered 12 h apart prior to bronchoscopy-guided intrabronchial lipopolysaccharide (LPS) stimulation (control: saline into the contralateral lung segment). We quantified ACE2 concentration, the Angiotensin-II-to-Angiotensin-1-7 conversion rate as well as FDP in BALF 6 h after LPS instillation. In placebo-treated subjects, LPS instillation increased ACE2 concentrations compared to unstimulated lung segments [1,481 (IQR: 736–1,965) vs. 546 (413–988) pg/mL; *p* = 0.016]. Dexamethasone abolished the increase in ACE2 concentrations (p=0.13). Accordingly, LPS instillation increased the Angiotensin-II-to-Angiotensin-1-7 conversion capacity significantly in the placebo cohort, indicating increased enzymatic activity (*p* = 0.012). FDP increased following LPS-instillation [8.9 (2.7–12.2) vs. 6.6 (0.9–9.6) ng/mL, *p* = 0.025] in the placebo group, while dexamethasone caused a shut-down of fibrinolysis in both lung segments. LPS instillation increased ACE2 concentration, its enzymatic activity and FDP, which was mitigated by systemic dexamethasone treatment. Our results strengthen previously published findings regarding the efficiency of corticosteroids for the treatment of COVID-19-induced acute lung injury.

## Introduction

Corticosteroids are widely used as an anti-inflammatory treatment in lung disease and have emerged as an effective treatment strategy in severe Corona Virus Disease 2019 (COVID-19) (1). Pulmonary edema is present in both COVID-19-induced and other forms of acute respiratory distress syndromes (ARDS), as a consequence of local inflammation and increased vascular permeability. Additionally, the contribution of vascular dysfunction to the pathogenesis of COVID-19 was highlighted (2). Lipopolysaccharide (LPS), a component of Gram-negative bacteria, may be instilled locally to model acute pulmonary inflammation. We have previously shown that dexamethasone reduces systemic inflammatory responses, pulmonary capillary leak and coagulation activation following bronchial instillation of LPS, while having limited effects on pulmonary pro-inflammatory cytokines (3, 4).

Angiotensin Converting Enzyme 2 (ACE2), a central enzyme of the alternative Renin-Angiotensin-Aldosterone System (RAAS), degrades angiotensin II (AngII), a proposed mediator of tissue damage in acute lung disease (5). The Severe Acute Respiratory Syndrome Corona Virus-2 (SARS-CoV-2) uses ACE2 as entry receptor. Downregulation of ACE2 was observed in murine models of lung injury including infection with SARS-CoV-1 and pulmonary LPS stimulation (6, 7). It was subsequently hypothesized that downregulation of ACE2 may disturb the pulmonary and possibly the systemic RAAS, resulting in a certain form of severe lung injury compatible with COVID-19. This pathophysiological concept, however, is primarily based on murine models. Importantly, ACE2 has been identified as an interferon-inducible gene in humans, which contrasts its murine regulation (8). In line, we observed increased systemic ACE2 concentrations in patients with severe COVID-19 that were associated with elevated Interleukin-6 (IL-6) levels (9).

It is well-established that inflammation activates coagulation and fibrinolysis in bacterial pneumonia, the latter primarily mediated by tumor necrosis factor alpha (TNF-α) (10). In COVID-19, a high rate of pulmonary arterial thrombosis has been reported, and elevation of D-Dimer and fibrin degradation products (FDP) were identified as robust predictors of mortality (11).

In the light of the ongoing discussion on ACE2 in lung injury and the relevance of coagulation activation in COVID-19, we quantified ACE2, its enzymatic activity and FDP in bronchoalveolar lavage fluid (BALF) from a pulmonary inflammation model in healthy volunteers. We hypothesized that bronchial LPS instillation increases pulmonary ACE2 and FDP concentrations, which may be mitigated by systemic dexamethasone treatment.

## Materials and Methods

Between 07/2011 and 06/2012, a randomized, double-blind, placebo-controlled trial was performed in 24 healthy volunteers at the Department of Clinical Pharmacology, Medical University of Vienna, Austria (NCT01714427). The institutional ethics committee of the Medical University of Vienna approved the trial (EK531/2010), which was performed in accordance with the Declaration of Helsinki. This study was initially conducted to investigate the systemic and pulmonary activation of coagulation and the inflammatory response after pulmonary instillation of LPS in healthy human volunteers (3, 4). This is an ancillary analysis of samples generated in this trial. Written informed consent was obtained from all study participants before trial enrollment. In short, in- and exclusion criteria comprised non-smoking healthy volunteers with unremarkable medical history, physical examination and laboratory investigations during screening, as well as normal findings in baseline chest radiography, spirometry and a negative routine drug screening.

Details on study design have been reported previously (3). Subjects were randomized to receive two infusions of 40 mg dexamethasone or saline 12 h apart in a double-blind manner. Prior to the study start, staff not otherwise involved in the study created a randomization list using online randomization software (http://www.randomization.com). Based on this list, two sets of sealed, opaque envelopes were prepared, which were labeled with randomization numbers and contained information on the subject-specific treatment allocation. Eligible subjects were assigned a randomization number. To maintain the double-blind character of the trial, study staff not otherwise involved in the trial prepared the study drug based on the information derived from the sealed envelopes. Another set of envelopes was kept for safety reasons, in case unblinding of subjects was necessary. The investigational medicinal products were not distinguishable from each other based on their physicochemical properties. Subjects received the first infusion 13 h prior to the first bronchoscopy. The second infusion was administered 1 h before the start of the bronchoscopy. Subjects were pretreated with dihydrocodeine (Teofarma, Valle Salimbene, Italy). The first bronchoscopy was performed under sedation with midazolam and propofol, which were titrated to obtain the desired effects. Once the bronchoscope was placed in a subsegment (middle lobe or lingula) a balloon-tipped monitoring catheter (Swan-Ganz catheter, Edwards Lifesciences, Irvine, CA, USA) was inserted and inflated: Ten mL of prewarmed, isotonic saline and 10 mL of air were instilled. Thereafter, 4 ng/kg bodyweight LPS (National Reference Endotoxin, *Escherichia coli* O:113, CC-RE-Lot 3, NIH, dissolved in mL saline), 10 mL prewarmed, isotonic saline and 10 mL air were instilled into the contralateral lung. After 6 h, bilateral bronchoalveolar lavage (BAL) was performed in exactly the same locations. During BAL, a total of 140 mL prewarmed saline in aliquots of 20–40 mL were instilled into both lung segments. The retrieved volumes were comparable between both study drugs and lung sites (median retrieval was ~45–55 mL).

Vital signs including blood pressure, heart rate, oxygen saturation, and body temperature were closely monitored throughout the trial.

The supernatant of the BALF was obtained as previously described (3). BALF was put on ice after retrieval, centrifuged and the supernatant was aliquoted and stored at −80°C until analysis. Commercially available enzyme-linked immunoassays (ELISA) were performed to quantify concentrations of ACE2 (human ACE2 Elisa, MyBioSource MBS824839, San Diego, CA, USA) and fibrin degradation products (human FDP Elisa, ABclonal RK 01378, Woburn, MA, USA). Angiotensin II to Angiotensin 1-7 conversion rate in BALF was determined after spiking samples with Angiotensin II as natural substrate and subsequent incubation at 37°C in both the presence and the absence of the specific ACE2-inhibitor MLN-4760. Quantification of Angiotensin II and Angiotensin 1-7 was conducted using LC-MS/MS to calculate the ACE2-specific Angiotensin 1-7 formation rate (Attoquant Diagnostics, Vienna, Austria).

A formal sample size calculation for the here presented exploratory analyses was not performed. The sample size was originally calculated with regards to prothrombin fragment F1+2 concentrations and interleukin-6 concentrations (3, 4). We present medians and quartiles. Furthermore, we present boxplots with whiskers (5–95% percentile). For reasons of robustness, two-group comparisons were performed by non-parametric Wilcoxon-Signed-Rank test or the Kruskal-Wallis test (as applicable). Due to the exploratory nature of the analyses, corrections for multiple testing were not conducted.

## Results

Nine women and 15 men were included in the trial. Due to a randomization error, 13 subjects received placebo, while 11 received dexamethasone.

Overall, no severe adverse events occurred, four subjects in the placebo group developed fever after the first BAL. Overall, eight subjects reported cough, three noted throat pain, while two subjects vomited. These results were already presented elsewhere, as two prior analyses have focused on the activation of coagulation and inflammation (3, 4).

### ACE2

**Figure 1:**
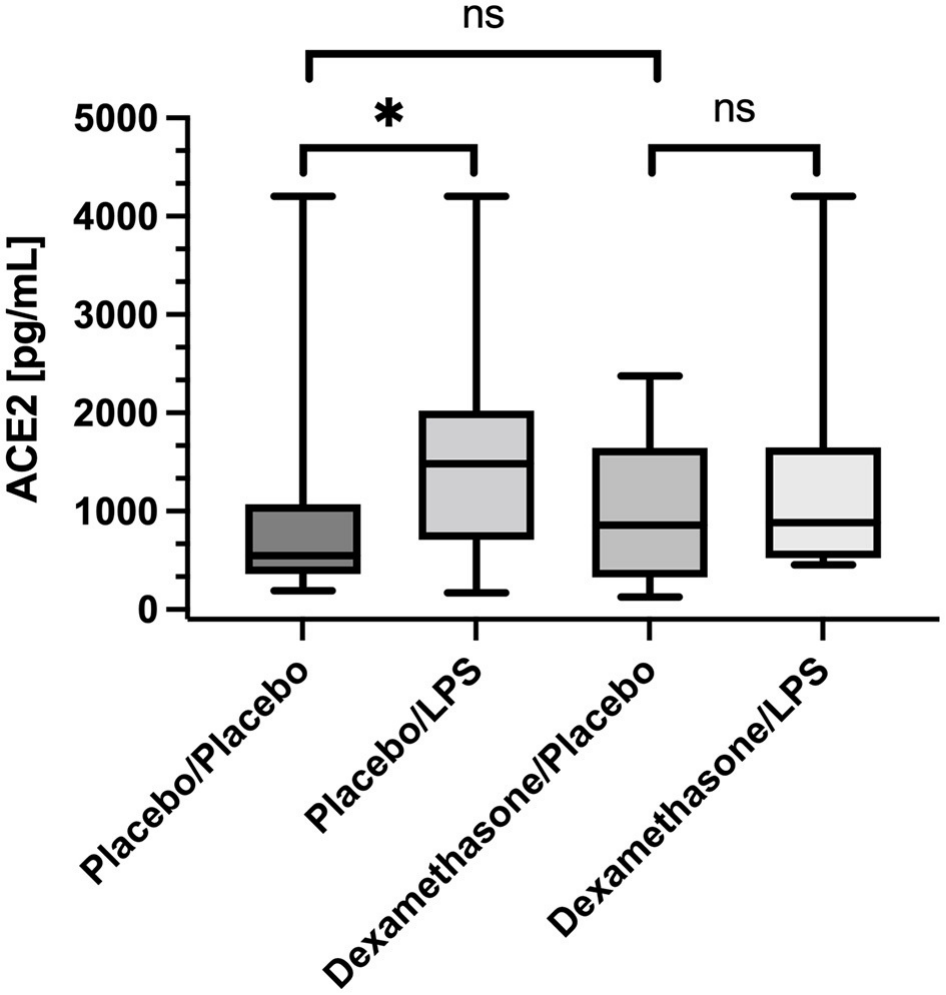
Angiotensin Converting Enzyme 2 (ACE2) concentration in Bronchoalveolar lavage fluid (BALF) samples. Lipopolysaccharide (LPS) instillation resulted in a significant increase in BALF ACE2 concentration (compared to the unstimulated contralateral side, *p* = 0.016) that could be mitigated by systemic application of dexamethasone (no significant difference). We present boxplots and whiskers (5–95% percentile). *N* = 24. ^*^ means statistically significant.

In placebo treated subjects, median ACE2 concentrations in BALF were ~3-fold higher in lung areas with local pulmonary LPS instillation compared to the contralateral, unstimulated lung areas [1,481 (736–1,965) vs. 546 (IQR 413–988) pg/mL, *p* = 0.016, (Figure 1)]. In contrast, dexamethasone pre-treatment, abolished the LPS-induced increase in ACE2 concentrations observed in placebo treated subjects [857 (326–1644) vs. 884 (522–1,649) pg/mL, *p* = 0.13, (Figure 1)]. Comparing ACE2 concentrations in unstimulated lung areas between individuals receiving placebo or dexamethasone did not show a statistically significant difference [546 (413–988) vs. 857 (326–1,644) pg/mL, *p* = 0.66], but suggests intraindividual variation in baseline ACE2 concentration in BALF from unstimulated lung segments.

**Figure 2:**
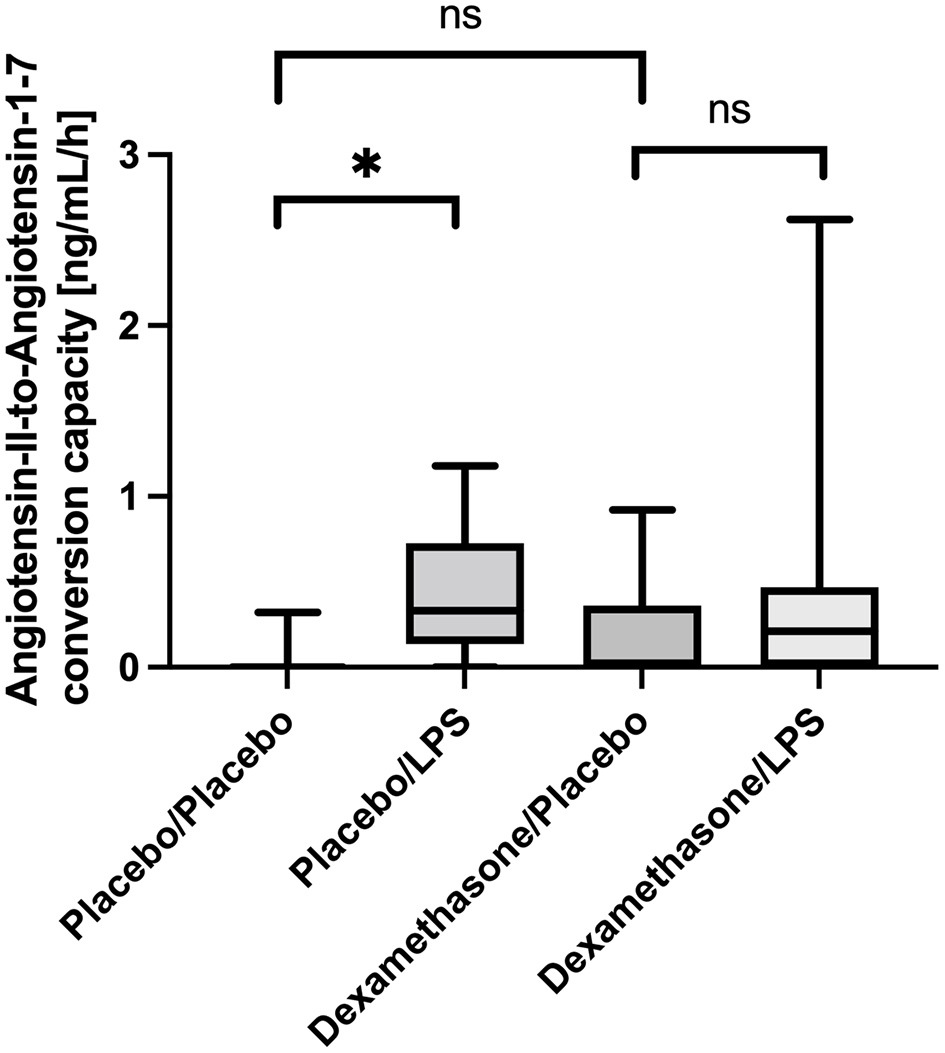
Angiotensin-II-to-Angiotensin-1-7 conversion capacity in ng/mL/h Bronchoalveolar lavage fluid (BALF) samples. Lipopolysaccharide (LPS) instillation resulted in a significant increase in the Angiotensin-II-to-Angiotensin-1-7 conversion capacity compared to the unstimulated lung site (*p* = 0.012) in placebo treated healthy volunteers. No significant difference was observed in dexamethasone treated subjects between LPS-stimulated and control lung sites. In unstimulated lung sites, the capacity was mostly under the lower-limit of detection of the applied assay. We present boxplots and whiskers (5–95% percentile). *N* = 24. ^*^ means statistically significant.

In line with this, we also found an increased capacity of Angiotensin-II-to-Angiotensin-1-7 conversion capacity in BALF from LPS stimulated lung segments in patients receiving placebo: In unstimulated lung segments the median ACE2-dependent Ang1-7 production capacity was at the lower level of quantification (LLOQ), while following LPS stimulation Angiotensin 1-7 production capacity increased to 26 (22–55) ng/mL/h, *p* = 0.012 (Figure 2). In contrast, in dexamethasone treated subjects no difference in Angiotensin 1-7 conversion was found between the LPS stimulated lung side vs. the control [LLOQ (LLOQ-28) vs. 17 (LLOQ vs. 37) ng/mL/h, *p* =0.26]. Infusion of dexamethasone did not change Angiotensin 1-7 production capacity in unstimulated lung segments compared to placebo (*p* = 0.15). Correlation between ELISA based quantification of ACE2 protein concentration and the LC-MS/MS based quantification of the Angiotensin-II-to-Angiotensin-1-7 conversion capacity was poor (*R* = 0.175; *p* = 0.15).

### Fibrin Degradation Products

**Figure 3:**
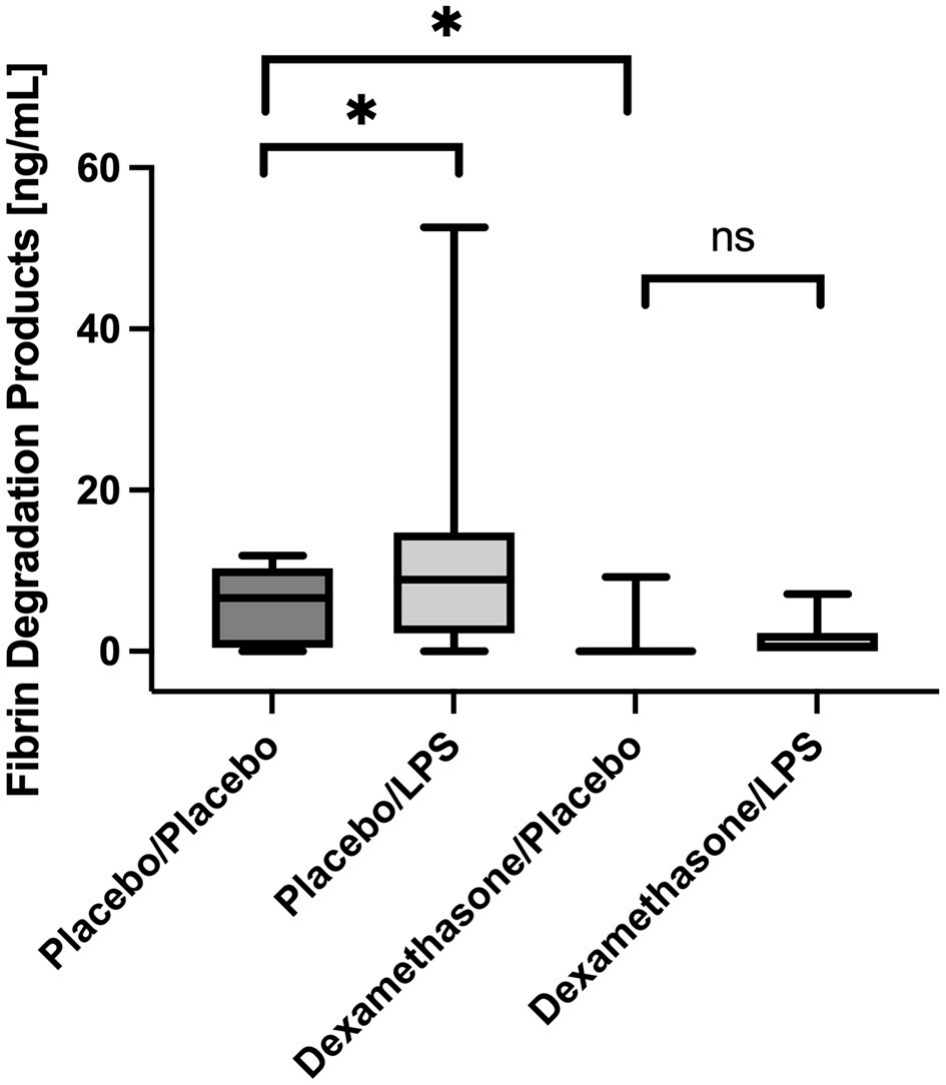
Fibrin degradation products (FDP) in Bronchoalveolar lavage fluid (BALF) samples. In placebo treated subjects, FDP concentrations were higher in BALF obtained from LPS stimulated lung segments compared to contralateral controls (*p* = 0.025). Infusion of dexamethasone resulted in an almost immediate shut-down of fibrinolysis in both stimulated and unstimulated lung segments. No significant increase was observed after LPS stimulation. We present boxplots and whiskers (5–95% percentile). *N* = 24. ^*^ means statistically significant.

In placebo-treated patients FDP concentrations were higher in BALF obtained from LPS stimulated lung segments compared to the contralateral controls [8.9 (2.7–12.2) vs. 6.6 (0.9–9.6) ng/mL, *p* = 0.025, (Figure 3)]. However, infusion of dexamethasone resulted in an almost complete shut-down of fibrinolysis in both lung sides [LLOQ (LLOQ-LLOQ) vs. 0.6 (LLOQ-2.3) ng/mL; *p* = 0.25]. In line, dexamethasone reduced FDP concentrations in the unstimulated lungs when compared to placebo [LLOQ (LLOQ-LLOQ) vs. 6.6 (0.9-9.6) ng/mL, *p* = 0.005].

## Discussion

Endotoxin-induced lung inflammation increased ACE2, its enzymatic activity and FDP in BALF of healthy volunteers, which was mitigated by dexamethasone. The observed increase in enzymatically active ACE2 in BALF is in contrast to murine models showing decreased ACE2 concentrations in BALF following LPS or virus-induced lung injury (6, 7). We applied two independent methods to quantify ACE2 protein concentration (ELISA) as well as ACE2-dependent enzymatic activity (Angiotensin-II-to-Angiotensin-1-7 conversion capacity).

In animal models, deficiency of ACE2 enzymatic activity drives lung injury by impaired degradation of angiotensin II (5, 6). In line with this, higher levels of ACE2 were found to be protective in various models of lung injury. Only recently ACE2 was identified as an interferon-inducible gene in humans. This is in contrast with murine data (8).

Onabajo et al. suggested that interferon induces a truncated form of ACE2, not serving as SARS-CoV-2 receptor and lacking endopeptidase activity (12). However, we demonstrated that both ACE2 concentration and enzymatic activity increased in BALF following LPS instillation. Low correlation of ELISA-based quantification of protein concentration and the enzymatic activity may reflect the increase of both active full-length and truncated ACE2. A higher rate of shedding of membrane-bound ACE2 and upregulated gene expression are both plausible mechanisms for the observed increase in BALF ACE2 concentration. Gene expression data in COVID-19 patients showed a ~200-fold increase in ACE2 expression levels in BALF cells (13). In line with this, systemic levels of enzymatically active ACE2 increased in severe COVID-19 patients compared to less severe cases, and correlated with systemic IL-6 levels (9). The observed mitigation of ACE2 by steroid treatment further supports an inflammation-driven upregulation of ACE2 in humans.

Dexamethasone caused an almost complete shutdown of fibrinolysis (10). It also reduced intrapulmonary prothrombin fragments, which is intriguing since glucocorticoid treatment reduced plasma levels of TNF-α and IL-6 during human endotoxemia, but did not affect LPS-induced activation of coagulation systemically (14). It is possible that dexamethasone reduces pulmonary permeability, which was shown by reduced BALF concentrations of immunoglobulins, as well as a reduced migration of inflammatory cells (3). Since histopathologic studies in deceased COVID-19 patients reported intra-alveolar fibrin deposition, these findings may further support a beneficial role of dexamethasone treatment (10).

Taken together, our data shows that ACE2 increases in human lung inflammation. The apparent species difference in ACE 2 regulation may have important implications for the current pathophysiological understanding of lung disease. Dexamethasone reduced ACE2 upregulation and intra-alveolar markers of fibrinolysis, which, in combination with the previously shown reduced activation of coagulation, may support its beneficial role in pulmonary diseases including, but not limited to, COVID-19.

## Data Availability Statement

The raw data supporting the conclusions of this article will be made available by the authors, without undue reservation.

## Ethics Statement

The studies involving human participants were reviewed and approved by the Institutional Ethics Committee of the Medical University of Vienna approved the trial (EK531/2010). The patients/participants provided their written informed consent to participate in this study.

## Author Contributions

RR-S, FE, and CS designed the study, analyzed the data, and wrote the manuscript. CS performed statistical analysis. JB, BJ, AH, and MH supervised the study and critically read and reviewed the manuscript. All authors contributed to the article and approved the submitted version.

## Funding

This work was supported by the Austrian Science Fund, FWF, grant KLI 861-B and by the Medical-scientific Fund of the Mayor of the Federal Capital Vienna, grant MA 40-GMWF COVID027.

## Conflict of Interest

The authors declare that the research was conducted in the absence of any commercial or financial relationships that could be construed as a potential conflict of interest.

## Publisher's Note

All claims expressed in this article are solely those of the authors and do not necessarily represent those of their affiliated organizations, or those of the publisher, the editors and the reviewers. Any product that may be evaluated in this article, or claim that may be made by its manufacturer, is not guaranteed or endorsed by the publisher.
